# Shielding the First 24 Postnatal Months of Life: A Proposal for a Prospective Cohort Study of Early-Life Electromagnetic Exposure and Autism Risk

**DOI:** 10.31083/AP53282

**Published:** 2026-08-24

**Authors:** Alberto Garoli, Alessandro Greco

**Affiliations:** ^1^Department of Morphology, Surgery and Experimental Medicine, University of Ferrara, 44121 Ferrara, Italy; ^2^Medical Direction, APSP (Public Agency for Personal Health Services), 38023 Cles, Italy; ^3^Course Direction, RSA (Residential Care Home) Medical Training Course for the Province of Trento, 38122 Trento, Italy; ^4^Research and Development Department, S.I.S.T.E.M.I. srl, 38057 Pergine Valsugana, Italy

**Keywords:** autism spectrum disorder, electromagnetic fields, neurodevelopment, mirror neuron system, ion cyclotron resonance, gene-environment interaction

## Abstract

**Background:**

Autism Spectrum Disorder (ASD) involves Mirror Neuron System (MNS) dysfunction, driving core social and imitative impairments. Systemic physiological alterations such as autonomic dysregulation, mitochondrial dysfunction and neuroinflammation are known to impair synchronization and plasticity of neuronal clusters. A less-evident environmental cofactor, coinciding with rising ASD prevalence, is the considerable world-wide increase in electromagnetic radiation (EMR) overall exposure among children. Experimental evidence shows how low-intensity EMR influences cellular processes, via voltage-gated calcium channels (VGCCs), oxidative stress, and mitochondrial metabolism. The Resonant Convergence framework, allow to predict how chronic EMR exposure during the first 24 postnatal months of life can act as a factor in ASD pathogenesis. The best candidate mechanism is chronic Ion Cyclotron Resonance (ICR) detuning the Ca^2+^-calmodulin pathway, thus disrupting MNS synchronization.

**Methods and Analysis:**

A prospective observational pilot cohort study (24-month follow-up) proposes to enroll 1000 full-term newborns into two arms: an EMR-reduced cohort (n = 500, rest and sleep-phase Faraday shielding) and a standard exposure cohort (n = 500). Exposure is quantified via radiofrequency (RF)/extremely low frequency(ELF) measurements, proximity analysis, device inventories and wearable dosimetry. The primary endpoint is a continuous neurodevelopmental trajectory score (joint attention, language, electroencephalogram (EEG) mu-rhythm); binary ASD diagnosis (Autism Diagnostic Observation Schedule, Second Edition (ADOS-2), Autism Diagnostic Interview-Revised (ADI-R)) is a secondary, exploratory endpoint. Moreover, an optional genomic screening will evaluate gene-environment interactions within extremely low-frequency electromagnetic field (ELF-EMF) vulnerable pathways, including ASD-associated genes upregulated by RF via bromodomain and extraterminal protein (BET)-mediated epigenetic mechanisms. Analyses will employ risk ratios, Fisher’s exact tests and logistic regression adjusted for confounders; mixed-effects and Bayesian modeling will evaluate longitudinal outcomes and exposure reduction effects. Given a 2–3% baseline prevalence, approximately 20–30 ASD cases are expected. The study is therefore powered for exploratory signal detection rather than definitive causal inference, providing the critical baseline data required to justify and design future confirmatory trials. Sex-stratified modeling will address the 4:1 male-to-female prevalence ratio.

**Ethics and Dissemination:**

Ethics committee approval is not yet sought; full protocol review and approval will be obtained prior to the study initiation, in strict accordance with the Declaration of Helsinki. Written parental informed consent will be mandatory for all participants prior to enrollment. Study findings and methodological milestones will be disseminated through peer-reviewed international scientific publications. This protocol provides a structured methodological framework for the first prospective investigation of sleep-phase EMR reduction as a potential modulator of ASD incidence during early neurodevelopment. Results will inform adequately powered confirmatory trials in electromagnetic neurodevelopmental epidemiology.

## 1. Introduction

Autism Spectrum Disorder (ASD) is a heterogeneous neurodevelopmental condition characterized by impairments in social communication, restricted behaviors, and atypical sensory processing. Despite extensive research, the underlying neurobiological mechanisms responsible for ASD remain incompletely understood. Increasing evidence suggests that disruptions in the development of neural circuits responsible for social cognition occur early in life and may involve both genetic and environmental influences [1,2]. Building on these observations, the present work proposes an integrative framework linking electromagnetic environmental exposure, mitochondrial-redox imbalance and altered neural synchronization in the developing brain, examining how disruptions in calcium-dependent signaling and thermodynamic homeostasis could, in fact, impair mirror-neuron-dependent networks, critical for social cognition. The convergence of genetically inherited electrophysiological sensitivity, neurodevelopmental vulnerability and strongly increasing environmental exposure warrants rigorous investigation.

### 1.1 Mirror Neuron System Dysfunction in ASD

As highlighted in our previous work [3], Mirror Neuron System (MNS) hypoactivation during social tasks is well documented in ASD [4,5,6,7]. Building on the pioneering discoveries of Prof. Giacomo Rizzolatti and his research team at the University of Parma (Italy), the first mirror neuron system was identified in the early 1990s [8,9,10]. These specialized neural circuits are now recognized as providing a fundamental biological substrate for imitation, action understanding, empathy, intention recognition and social cognition. Subsequent human neuroimaging and neurophysiological studies have demonstrated that dysfunction within the mirror neuron network contributes to many of the core social-communication deficits observed in Autism Spectrum Disorder (ASD), making these circuits a biologically plausible target for specialized analyses [5,7,10]. Consistent with this rationale, our previous pilot study demonstrated improvements across multiple dimensions of ASD symptomatology, following targeted extremely low-frequency electromagnetic field (ELF-EMF) treatment. Our previous study, although still leveraging a small sample, supports the hypothesis that these interventions may enhance functional organization and synchronization of dysregulated neural networks, rather than compensate for irreversible structural brain injury [3].

Neuroimaging and electrophysiological investigations have consistently shown that ASD is characterized by atypical patterns of functional and structural connectivity, including local hyperconnectivity, reduced long-range connectivity, altered network synchronization, and impaired predictive coding mechanisms [11,12,13]. Importantly, these abnormalities reflect an altered trajectory of early brain development rather than acquired inflammatory destruction of neural tissue. One of the most reproducible findings in autism research is early brain overgrowth, occurring during infancy and the first years of life. This is characterized by accelerated expansion of total brain volume, increased cortical thickness, enlarged cortical surface area, and increased grey matter volume in several cortical and subcortical regions. These developmental changes are believed to arise from altered neurogenesis, abnormal synaptic pruning, atypical dendritic arborization, and disrupted maturation of neuronal circuits. As development progresses, these anatomical differences follow heterogeneous trajectories across individuals, with some regions partially normalizing while others continue to display atypical connectivity and cortical organization throughout life.

Furthermore, systemic physiological alterations — including autonomic dysregulation, mitochondrial dysfunction, oxidative stress, and neuroinflammation — have increasingly been recognized as contributing factors in ASD [14,15,16]. Recent studies of autonomic nervous system (ANS) function suggest that many individuals with ASD exhibit autonomic dysregulation, most commonly characterized by reduced vagally mediated heart rate variability (HRV), reflecting diminished parasympathetic regulation and impaired autonomic adaptability [14,17]. Such alterations may contribute to a reduced capacity to dynamically adjust physiological responses to changing internal and external environmental demands. At cortical level, electroencephalogram (EEG) coherence and functional connectivity analyses have identified reproducible electrophysiological signatures that distinguish ASD from neurotypical brain organization [18]. These findings are consistent with the presence of atypical large-scale neural network synchronization and altered oscillatory connectivity [8,9,19,20], phenomena that are thought to be caused by atypical neurodevelopment and neuroimmune mechanisms [1,16].

Among contributing factors in ASD, mitochondrial dysfunction has emerged as a significant factor in a substantial subset of affected individuals. Reduced activity of mitochondrial electron transport chain complexes I and IV, accompanied by increased production of reactive oxygen species (ROS), has been reported in multiple studies, indicating impaired oxidative phosphorylation and increased oxidative stress [15]. Together, these abnormalities reduce cellular bioenergetic efficiency and compromise neuronal homeostasis. From a nonequilibrium thermodynamic perspective, these observations may also be interpreted within the framework proposed by Lucia [21,22,23], in which living systems continuously dissipate energy while maintaining ordered functional states. According to this model, impaired mitochondrial energy metabolism and excessive oxidative stress could reduce the capacity of neuronal networks to sustain efficient entropy production and dynamic self-organization. Within highly integrated systems such as the mirror neuron network (MNS), this condition is hypothesized to impair large-scale oscillatory synchronization and phase coherence required for efficient information processing. Although this thermodynamic interpretation remains a theoretical framework requiring further experimental validation, it provides a first coherent biophysical model linking mitochondrial dysfunction, oxidative stress, altered network synchronization, and the behavioral manifestations characteristic of ASD.

### 1.2 Neural Network Development and the Critical Vulnerability Window

Early childhood — especially the window between 6 and 18 months — is a critical period for the maturation of the MNS and, in general, grey matter [24,25,26,27]. During this developmental phase, infants undergo rapid changes in: imitation ability [25], joint attention [24], eye-gaze synchronization [24], early language acquisition [24], emotional resonance and social referencing [26]. All of these behaviors depend on tightly synchronized interactions between visual perception, motor planning, and limbic regulation — domains strongly overlapping with MNS-related circuits in the inferior frontal gyrus, inferior parietal lobule, and superior temporal sulcus [24,25]. Because these networks rely on precise spike-timing-dependent plasticity and oscillatory synchronization, they are particularly vulnerable to disturbances in neural timing and connectivity during early development [13].

Altered connectivity between MNS regions and other brain areas has been shown to disrupt the integration of social information [2,12]. Very importantly for the sake of our study, Dapretto et al. [6] demonstrated reduced activation in the Inferior Frontal Gyrus (IFG), a key mirror neuron region, during imitation tasks in children with ASD compared to typically developing controls. At the oscillatory level, the most common findings include: reduced delta amplitude [4] and reduced oscillatory coherence in the mu rhythm (8–13 Hz). This last phenomenon is a typical electrophysiological signature associated with mirror neuron activation [4,6]. Moreover, impairment in the formation of predictive coding loops is evident. These phenomena appearing in sensorimotor cortices prevent infants from anticipating other people’s actions and intentions [13]. Lastly, reduced activation of the IFG might produce errors in social reward timing, making eye contact and shared attention less reinforcing [28].

The MNS depends on the precise temporal coordination of motor and perceptual networks, suggesting that both its structural organization and functional information processing rely on tightly regulated time-dependent mechanisms [29]. During early development, the MNS exhibits a high degree of plasticity as motor and perceptual systems undergo rapid maturation, requiring continuous calibration and functional refinement. This developmental period likely represents a window of increased vulnerability, during which even subtle perturbations in temporal coordination may interfere with normal maturation [30]. Consequently, disruptions in the timing of neural activity during this critical developmental phase have been proposed as a plausible mechanism contributing to mirror neuron system dysfunction [31].

### 1.3 Anthropogenic EMR as a Putative Modulator of Connectome Development

In a prior study we observed that the human brain can be sensitive at intensities ranging 20–30 µT and within windows of 1–4 Hz [32]. Over the past two decades, ambient radiofrequency electromagnetic field (RF-EMF) exposure has increased substantially owing to the widespread deployment of mobile telecommunications, wireless networking technologies, and satellite communication systems [33]. During the same period, reported ASD prevalence has also risen markedly, although this temporal association should not be interpreted as evidence of a causal relationship, given the substantial contributions of evolving diagnostic criteria, increased awareness, and other genetic and environmental factors [34]. Nevertheless, very recent experimental findings have identified biologically plausible mechanisms through which RF exposure may influence early neurodevelopment. In particular, a human cortical organoid study demonstrated that chronic RF exposure (800–2400 MHz) altered radial glial differentiation, delayed corticogenesis, and induced the expression of ASD-associated genes through BET (bromodomain and extraterminal protein)-mediated epigenetic pathways. Importantly, pharmacological inhibition of BET proteins substantially rescued these developmental abnormalities, supporting a mechanistic role for epigenetic regulation in the observed effects [35]. Although these findings originate from an *in vitro* developmental model and therefore cannot be directly extrapolated to human ASD, they provide compelling mechanistic evidence that RF-EMF exposure is capable of modulating molecular and cellular processes relevant to early corticogenesis, thereby warranting further investigation in translational and epidemiological studies.

Non-ionizing electromagnetic fields in the extremely low-frequency (ELF) and radiofrequency ranges are now ubiquitous in modern environments [36,37]. Experimental studies have reported that electromagnetic fields can influence biological systems through mechanisms involving voltage-gated calcium channels, mitochondrial metabolism, and oxidative stress pathways [38,39,40]. In light of the neurobiological vulnerabilities inherent and characteristic of early childhood, we postulate that the chronic exponential increase in early exposure to environmental EMR might act as an etiological cofactor in the current rise of ASD incidence, disrupting delicate signal-driven, neurodevelopmental processes and the structuring of cortical networks.

While correlation does not establish causation, the marked rise in the anthropogenic electromagnetic background warrants rigorous biophysical evaluation of its potential neurodevelopmental impacts, particularly due to the intrinsic sensitivity and vulnerability of the emerging infant connectome. Grey matter development accelerates between 6 and 18 months of age [24,25,26]. Experimental models suggest that environmental EMFs may alter oligodendrocyte function or disrupt the oxidative stress balance, potentially influencing conduction velocity — and consequently precise neural timing — across developing cortical networks, including the MNS [2,16]. Moreover, the marked 4:1 male-to-female prevalence ratio in ASD incidence [41] may be linked to a differential neurobiological responsiveness of grey matter to specific EMR signals during early windows of development. Elucidating the neurodevelopmental effects of environmental electromagnetic radiation (EMR) requires a mechanistic understanding of the biophysical processes through which electromagnetic fields interact with the developing nervous system. Defining these interaction pathways is essential for establishing biologically plausible mechanisms linking EMR exposure to alterations in neural development.

### 1.4 Biophysical Mechanisms: VGCC Activation, Ca^2+^ Overload, and the Mitochondrial-Microglial Axis

The developing brain exhibits intrinsic oscillatory frequencies (delta, theta, alpha, gamma) [42]. It has been hypothesized that temporally modulated EM signals could entrain or interfere with endogenous neural rhythms through resonance phenomena [43]. While robust macroscopic resonance at environmental RF intensities remains debated *in vivo* due to tissue shielding and thermal regulation [44,45], the biological impact of low-intensity EMR has been empirically documented across multiple domains, including motor tremor [46], reaction time alterations [47], sleep pattern disruptions [48], and indirect EEG modulation [49,50], as well as broader developmental outcomes [51]. Consequently, environmental EMFs represent a critical exogenous trigger for developing neural networks. Because MNS maturation relies strictly on precise spike-timing-dependent plasticity (STDP) [52], alpha/mu synchronization [53], and experience-dependent calibration [54,55], any environmental factor modulating these calcium-dependent learning processes can theoretically interfere with early social-cognitive circuitry [35,56]. In accordance with the Resonant Convergence model, the primary biophysical transducer that explains these diverse EMR-induced alterations is the voltage-gated calcium channel (VGCC) [57]. The modulation of VGCCs by external electromagnetic fields occurs through the alteration of their gating probability, which directly impacts intracellular Ca^2+^ flux [39]. A minor depolarization bias can initially amplify physiological signaling cascades, increasing N-methyl-D-aspartate (NMDA) receptor sensitivity and the activation of cAMP response element-binding protein (CREB) — a crucial transcription factor that regulates the expression of genes involved in synaptogenesis and synaptic plasticity [58]. However, sustained or anomalous activation by low-intensity EM fields leads to deleterious intracellular Ca^2+^ elevation. Crucially, this EMR-induced calcium overload is strictly transduced through the Ca^2+^-calmodulin (CaM) complex [57]. As a primary intracellular sensor, the Ca^2+^/CaM complex activates downstream effectors including calcium/calmodulin-dependent protein kinase II (CaMKII). This kinase is fundamental for synaptic scaling and the long-range phase-locking of the MNS [57]. Under the Resonant Convergence framework, the ICR-detuning of the Ca^2+^/CaM pathway is hypothesized to be the critical biophysical node disrupted by anthropogenic EMR, leading to the theoretically predicted electrodynamic decoupling.

Additionally, this excessive calcium influx triggers a well-documented pathological cascade: the cellular attempt to buffer excess Ca^2+^ leads to massive mitochondrial uptake, which in turn uncouples the respiratory chain and generates a surge in Reactive Oxygen Species (ROS) [40]. Calcium signaling—mediated by both CaM-dependent pathways and mitochondrial buffering—is the central bidirectional mediator of synaptogenesis and neuroinflammation. Consequently, an anomalous and continuous influx of Ca^2+^ during critical neurodevelopmental windows could theoretically bias synaptic stabilization, permanently altering the emerging network topology [40].

A specific mechanistic framework linking environmental exposure to disrupted connectome architecture can be established through the mitochondrial-redox and microglial axes [15,16]. Theoretical models indicate that low-intensity EMFs induce weak alternating electric fields within biological tissues [21,22,23]. This relationship can be approximated by the induction Eqn. 1:


(1) $$E_{\text{induced}}\approx 2\pi \cdot f\cdot B\cdot r$$

where f is the frequency, B is the magnetic flux density, and r is the effective cellular radius (~10^-5^ m). At geomagnetic orders of magnitude (~50 µT) and extremely low frequencies (10–100 Hz), the induced electric field is approximately 3.1 × 10^-8^ to 3.1 × 10^-7^ V/m [21]. Multiplying this induced field by the cellular radius yields an estimated transmembrane induced voltage of approximately 3.1 × 10^-13^ to 3.1 × 10^-12^ V — roughly nine to ten orders of magnitude below the ~10 mV depolarization threshold required for neuronal excitation. This magnitude gap demonstrates that direct, Faraday-induction-driven membrane depolarization is not quantitatively viable as a transduction mechanism at these field strengths. Within the Resonant Convergence framework, this gap is addressed not by direct depolarization but by a cascade-amplification mechanism, in which sequential voltage-gated channel avalanches and calmodulin-dependent enzymatic steps transform a sub-threshold perturbation into a macroscopic cellular response [57], consistent with the VGCC gating-probability mechanism described above [39].

Furthermore, experimental models have consistently reported increased ROS following EMF exposure [38,59,60]. EMF-driven intracellular Ca^2+^ elevation forces mitochondrial buffering via the mitochondrial calcium uniporter (MCU) [39,61]. This overload severely compromises mitochondrial bioenergetics, leading to impaired adenosine triphosphate (ATP) production, lipid peroxidation, and a surge in superoxide generation [15,40]. Clinically, this aligns with findings in ASD cohorts, which frequently exhibit elevated oxidative stress markers, altered glutathione ratios, and mitochondrial dysfunction phenotypes [15].

**Fig. 1.**
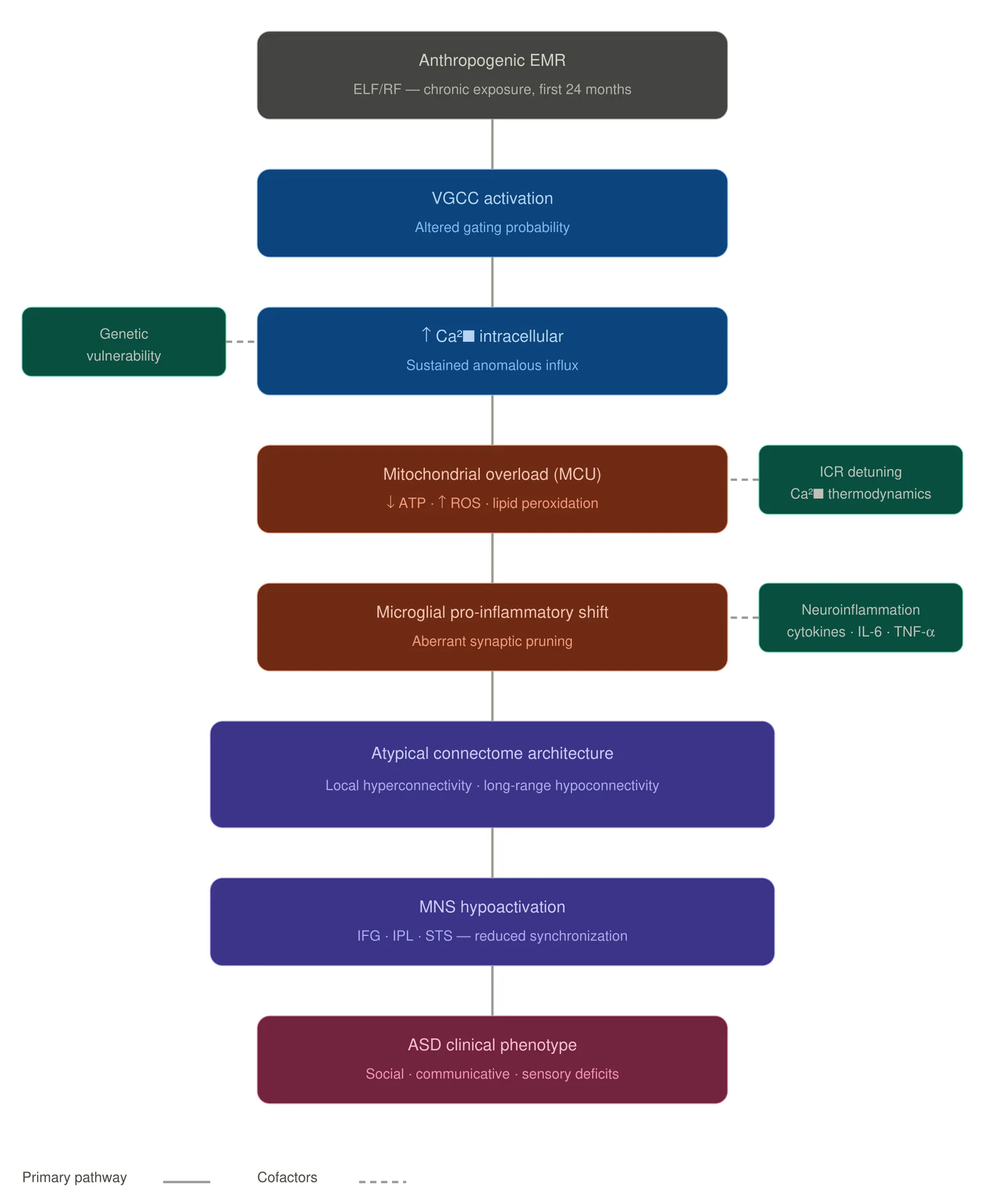
**EMR-ASD pathogenetic cascade**. Stepwise pathogenic cascade linking chronic EMR exposure to ASD phenotype through VGCC activation, mitochondrial-redox failure, and connectome alteration. EMR, electromagnetic radiation; ASD, Autism Spectrum Disorder; VGCC, voltage-gated calcium channel; ICR, Ion Cyclotron Resonance; MNS, Mirror Neuron System; IFG, Inferior Frontal Gyrus; IPL, Inferior Parietal Lobule; STS, Superior Temporal Sulcus; IL-6, Interleukin-6; TNF-α, Tumor Necrosis Factor-alpha; ROS, reactive oxygen species; ELF, extremely low frequency; RF, radiofrequency; MCU, mitochondrial calcium uniporter; ATP, adenosine triphosphate.

Because the developing brain is exceptionally plastic and relies on tightly regulated bioelectrical activity to guide its maturation, relatively small perturbations occurring during critical developmental windows may exert cascading effects on neural network formation [2]. Early corticogenesis depends on precisely coordinated endogenous bioelectrical signaling—including membrane potential dynamics, calcium transients, and spontaneous neuronal activity—to regulate neuronal proliferation, migration, differentiation, activity-dependent synaptogenesis, and the emergence of synchronized oscillatory networks, including theta–gamma coupling [42,62]. Within the mechanistic framework proposed herein, mitochondrial dysfunction and the associated increase in reactive oxygen species (ROS) are hypothesized to promote a shift in microglia toward pro-inflammatory activation states. Given that microglia are the principal mediators of developmental synaptic pruning, their dysregulation could disrupt the normal refinement of cortical circuits, thereby altering cortico-cortical connectivity gradients and the maturation of long-range association pathways [2,16]. As schematically illustrated in Fig. 1, this sequence provides a biologically plausible mechanistic model linking environmentally induced mitochondrial redox imbalance with altered microglial function and aberrant synaptic remodeling. Together, these processes are consistent with the atypical structural and functional connectome described in ASD, characterized by local hyperconnectivity alongside reduced long-range connectivity [2,12]. Although each step of this proposed cascade requires further experimental validation, the individual biological processes involved are independently supported by an extensive body of developmental neuroscience, neuroimmunology, and mitochondrial biology. Together, they provide a coherent pathogenetic framework amenable to direct experimental testing.

### 1.5 Resonant Convergence: ICR Detuning and Thermodynamic Failure

To unify the impact of low-intensity EMR, developmental anomalies in grey matter, and the mitochondrial-microglial cascade, we propose that ASD should be fundamentally interpreted as a pathology of electrodynamic tuning and thermodynamic coherence, according to the Resonant Convergence model [57].

Maintaining precise neural timing and long-range synchronization — such as the specific phase-locking required by the MNS — relies on highly synchronized transmembrane ion fluxes. According to the Ion Cyclotron Resonance (ICR) model [63,64,65,66], the physiological movement of key ions, particularly Ca^2+^, is only partially concentration-dependent and is instead highly tuned by endogenous electromagnetic fields. This tuning ensures the ultra-fast synaptic responses and network oscillatory entrainment required for instantaneous social referencing.

**Fig. 2.**
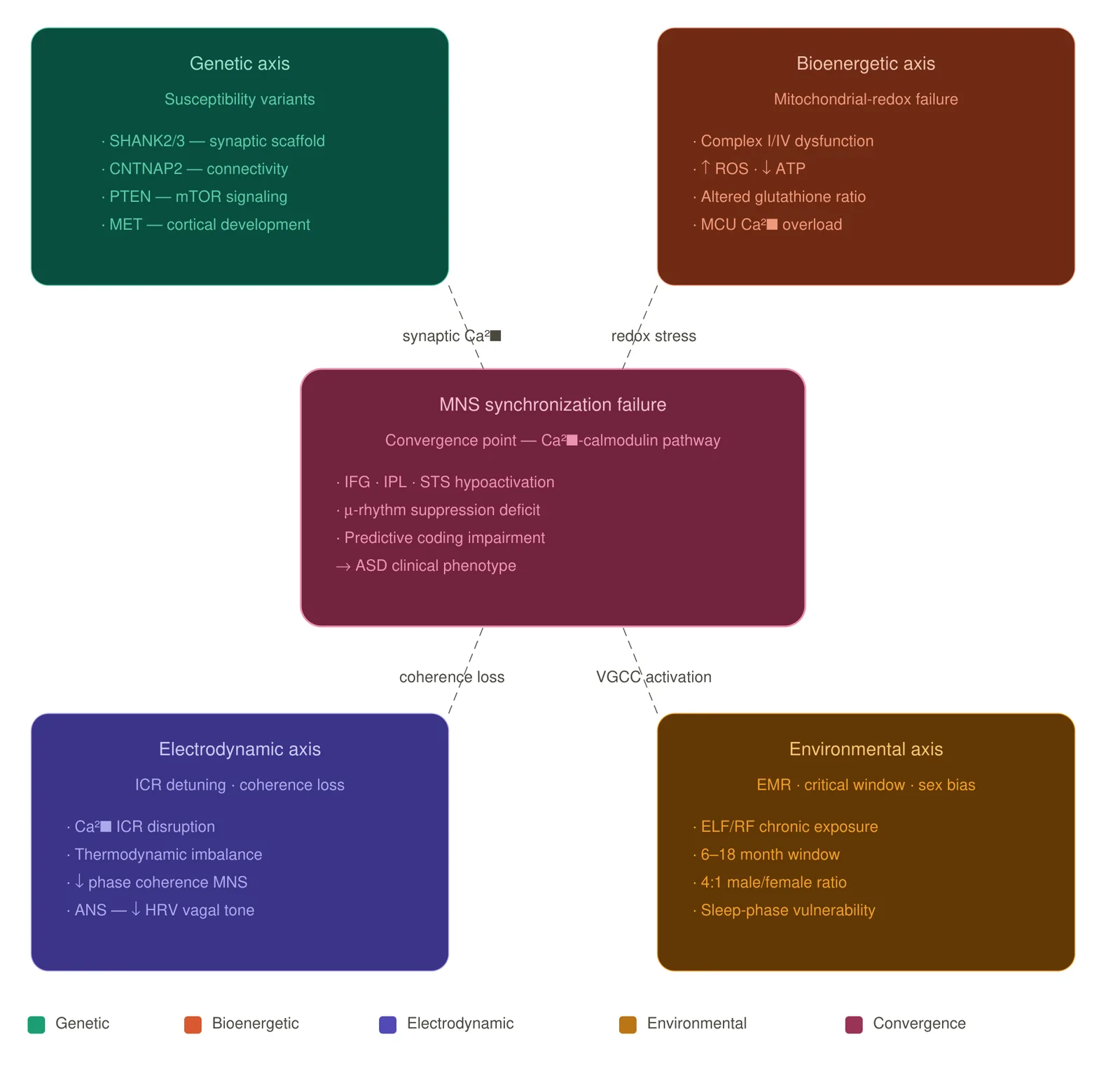
**Resonant convergence multiaxis model**. Resonant Convergence integrative model: four convergent pathogenic axes — genetic, bioenergetic, electrodynamic, and environmental — converging on Ca^2+^-calmodulin-mediated MNS synchronization failure as the common pathophysiological node in ASD. ANS, autonomic nervous system; HRV, heart rate variability; mTOR, mechanistic target of rapamycin.

As formalized in Eqn. 1, these exogenous low-frequency fields act as continuous perturbations on the delicate ICR tuning of transmembrane Ca^2+^ flux, which the Resonant Convergence model links to Ca^2+^/calmodulin- and CaMKII-dependent MNS phase-locking [57]. Within this framework, loss of resonant coherence is further conceptualized through a nonequilibrium thermodynamic lens linking cellular energy dissipation to electromagnetic responsiveness [21,22,23] (full formalism in **Appendix A.1**): the metabolic collapse and ROS accumulation described above are postulated to drive affected neurons into a state of entropic ‘thermodynamic chaos’, chronically distorting the endogenous magnetic microenvironment required for physiological ICR tuning (Fig. 2). This mechanism confronts a well-established theoretical objection — the incompatibility between weak-field Lorentz forces and physiological thermal noise (kT), first formalized by Adair [67,68,69] — which is explicitly acknowledged, not dismissed. ICR and TR are treated throughout this protocol as candidate, Tier-3 frameworks; the full thermal-noise and quantum-coherence-domain debate is presented in **Appendix A.2**. Critically, the protocol’s epidemiological objective is decoupled from resolution of this unresolved transduction debate.

The MNS requires extremely rapid, coherent phase-locking — particularly in the mu and gamma bands — to guarantee instantaneous imitation and empathy [4,6,53]. We hypothesize that an initial disruption of calcium ICR tuning — induced by exogenous EMFs on developing networks — triggers a catastrophic cascade of mitochondrial dysfunction and oxidative stress [59,61]. This resulting redox imbalance acts on two converging fronts: structurally, it shifts microglia toward pathological synaptic pruning, altering the physical connectome [16]; electrodynamically, it generates an internal thermodynamic noise that irreversibly detunes the physiological Ion Cyclotron Resonance of the neuronal networks [1,11,15,16,18,21,22,63,64,65,66]. This dual structural and electrodynamic disconnection prevents the long-range phase-locking of the MNS, translating clinically into the core social, communicative, and cognitive deficits characteristic of ASD [5,11,18,31,57].

### 1.6 Study Rationale, Primary Hypothesis, and Objectives

Considering the evidence reviewed above, we advance the primary hypothesis that chronic anthropogenic EMR exposure during the first 2 years of life — particularly during sleep phases of heightened synaptic consolidation — constitutes a modulatory etiological cofactor in ASD pathogenesis, acting through ICR detuning of the Ca^2+^/CaM pathway and consequent impairment of MNS synchronization.

To empirically test this hypothesis, this prospective longitudinal pilot study focuses on the critical first 2 years of brain maturation. Rather than establishing direct causality at this stage, the operational objective is to evaluate whether a systematic reduction of EMR exposure during sleep phases can mitigate the incidence or severity of ASD. The study prospectively follows 1000 full-term newborns (gestational age ≥37 weeks) for 24 months. Infants with major congenital neurological abnormalities, severe genetic syndromes, or prolonged neonatal intensive care hospitalization (>14 days) are excluded.

The primary epidemiological objective—assessing whether systematic sleep-phase EMR reduction lowers ASD incidence or severity—is empirically testable independent of the eventual validation of the biophysical mechanisms. ICR and TR are invoked here as heuristic explanatory models for the hypothesis, not as prerequisites for the epidemiological test.

## 2. Materials and Methods

### 2.1 Study Design and Cohort

To evaluate the proposed association between early-life exposure to anthropogenic electromagnetic radiation (EMR) and atypical neurodevelopmental trajectories, we designed a prospective longitudinal cohort study comprising two parallel observational groups. The study focuses on the first two years of postnatal life, a fully autonomous critical period of rapid brain growth and synaptic network formation, with each participant followed prospectively for 24 months. While corticogenesis and radial glial differentiation occur predominantly in utero [35], ASD-related behavioral phenotypes typically become clinically detectable between 6 and 24 months [24,25,26]; initiating enrollment at birth also reduces confounding from maternal physiology and prenatal factors that cannot be adequately controlled for in this design.

The study population will consist of 1000 full-term newborns (gestational age ≥37 weeks). Infants with major congenital neurological malformations, recognized chromosomal or severe genetic syndromes associated with neurodevelopmental disorders, significant perinatal brain injury, or prolonged neonatal intensive care unit (NICU) hospitalization exceeding 14 days will be excluded. Following written informed parental consent, infants will be enrolled into one of two exposure cohorts: an EMR-Reduced cohort or a Standard Exposure cohort (500 participants per group). Cohort allocation will not be randomized; participants will instead be classified according to parental acceptance of the environmental EMR-reduction protocol. Randomization was judged impractical and ethically problematic in this setting: no credible sham-shielding procedure exists that would preserve blinding while genuinely varying EMR exposure, and mandating a household-level environmental intervention against parental preference over a 24-month period would likely produce high, systematically differential non-adherence, undermining any intention-to-treat interpretation. Allocation by parental acceptance instead preserves the observational nature of the study while enabling prospective comparison between differing, self-selected exposure conditions — a design limitation addressed directly through the confounding-control strategy detailed below.

### 2.2 Exposure Control and Environmental Monitoring

**Fig. 3.**
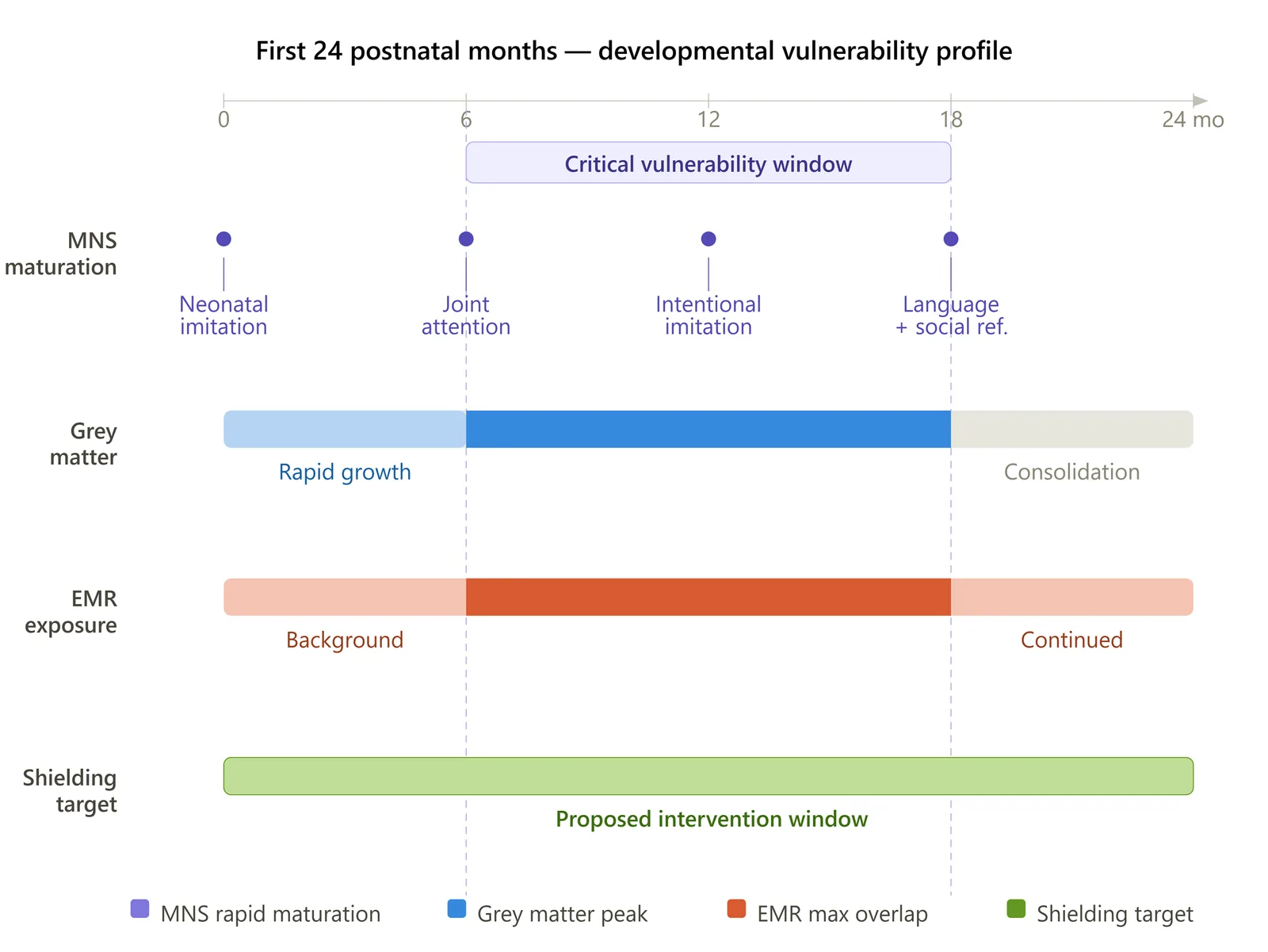
**Critical vulnerability window**. Temporal overlap between MNS maturation, grey matter acceleration, and EMR exposure during the first 24 months, highlighting the proposed sleep-phase shielding window.

The two observational cohorts are managed as follows:

• EMR-Reduced Cohort (n = 500): Subjects are raised under conditions designed to minimize electromagnetic exposure during rest and sleep, targeting the phases of highest synaptic consolidation (Fig. 3). Interventions include the installation of silver-coated, grounded Faraday canopy nets around sleeping areas, the removal of any wireless transmitting devices within the shielded space (e.g., Wi-Fi routers, baby monitors) and the preferential use of wired communication infrastructure in the room.

• Standard Exposure Cohort (n = 500): Subjects reside in typical modern residential environments without specific electromagnetic shielding interventions.

To ensure high-fidelity adherence within the EMR-Reduced cohort, the Faraday canopy nets will be equipped with embedded continuity sensors and radio-frequency identification (RFID) tags, providing objective, continuous shield-integrity monitoring. Study staff will provide standardized training to participating families on proper canopy installation and maintenance at enrollment, with structured in-home compliance verification visits conducted quarterly throughout the 24-month follow-up period. This automated technical data stream will be cross-validated on a monthly basis against parent-reported compliance logs and wearable dosimeter data, thereby minimizing reliance on self-reported metrics alone and establishing a multi-layered, objective exposure verification matrix.

### 2.3 Outcome Measures and Covariates

Because neurodevelopment during infancy is more sensitively characterized by continuous developmental measures than by a binary diagnostic outcome, the primary endpoint consists of a predefined composite continuous score integrating joint attention (Early Social Communication Scales; ESCS), early receptive and expressive language development (Communicative Development Inventories [CDI] and/or Mullen Scales of Early Learning [MSEL]), and electroencephalographic (EEG) mu-rhythm spectral power (8–13 Hz) [53]. This composite endpoint will be analyzed longitudinally using linear mixed-effects models, thereby maximizing statistical power while accounting for repeated within-subject measurements over the 24-month follow-up period.

A binary diagnosis of Autism Spectrum Disorder at 24 months, established according to ADOS-2, ADI-R, and Diagnostic and Statistical Manual of Mental Disorders, Fifth Edition (DSM-5) criteria, will constitute a prespecified secondary exploratory endpoint. Because the expected number of ASD cases is relatively small, this outcome will be analyzed using Firth’s penalized logistic regression to minimize small-sample and rare-event bias [70].

To estimate the independent association between EMR exposure and neurodevelopmental outcomes, while minimizing potential confounding arising from parental health-seeking behavior and other selection factors, multivariable regression models will be used. These will adjust for predefined covariates, including a standardized parental socioeconomic status index, objectively recorded child screen-time exposure, and baseline parental health anxiety assessed using the Short Health Anxiety Inventory (SHAI). Additional prespecified clinical and perinatal covariates will be incorporated where appropriate.

All neurodevelopmental assessments, including ADOS-2, ADI-R, and EEG acquisition and analysis, will be performed by trained evaluators blinded to cohort allocation in a standardized clinical environment designed to minimize inadvertent disclosure of exposure status. Video recordings and EEG datasets will be independently coded by assessors blinded to study arm, and inter-rater reliability will be formally evaluated and reported.

### 2.4 Genetic Susceptibility and Genomic Analysis

Baseline familial risk is documented through family history to distinguish genetically predisposed populations. Furthermore, the study incorporates an optional genomic screening component to evaluate Gene–Environment interactions. This screening targets candidate genes involved in biological pathways theoretically vulnerable to specific ELF-EMF modulation, including synaptic structure, neuroinflammation, circadian regulation, mitochondrial metabolism, and neurotransmitter signaling.

This gene-environment interaction framework is directly supported by recent experimental data demonstrating that RF exposure upregulates ASD-associated genes—including TBR1, ARX, FOXG1, and SCN2A—in human fetal cortical organoids via BET-mediated epigenetic mechanisms [35]. The broader candidate frequency-gene associations, with methodological details, full tables, and associated references, are moved to **Appendix A.3**. Within the Resonant Convergence framework, these associations are classified strictly as Tier-3 hypotheses—defined as biologically plausible and supported by preliminary observational data, yet lacking established, direct mechanistic confirmation in the human context [57]. Accordingly, the genomic screening component of this protocol is primarily exploratory and hypothesis-generating; it is not designed for causal inference, and the specific relationships hypothesized in **Appendix A.3** should not be construed as validated clinical or dosimetric guidance. As further detailed there, with the sole exception of the ADORA2A/3–75 Hz pairing—the only target supported by direct *in vitro* EMF evidence [71]—all other candidate frequencies represent theoretical extrapolations derived from the general ICR framework rather than gene-specific empirical findings.

### 2.5 Statistical Analysis and Power Considerations

Given the baseline ASD prevalence of 27.6 per 1000 (2.76%) among 8-year-old children in the United States [34], the sample size (n = 1000) is designed for feasibility and exploratory signal detection rather than definitive causal inference. The exact power profile and expected case numbers across varying relative risk reductions are detailed in Table 1.

**Table 1.** **Statistical power profile**.

Relative risk reduction	Expected cases (treatment vs. control, n = 500/arm)	Statistical power (Fisher’s exact, α = 0.05)
30%	~9.7 vs ~13.8	10.3%
50%	~6.9 vs ~13.8	27%
70%	~4.1 vs ~13.8	57.2%
80%	~2.8 vs ~13.8	75%

Expected case numbers in the control arm (standard exposure, n = 500) are calculated assuming a 2.76% baseline ASD prevalence (500 × 0.0276 ≈ 13.8 cases), consistent with the 27.6-per-1000 estimate reported among 8-year-old children in the US ADDM Network [34]. Statistical power evaluated via Fisher's exact test (α = 0.05, two-sided) across varying relative risk reductions. ADDM, Autism and Developmental Disabilities Monitoring.

The primary outcome, the composite trajectory score, will be evaluated via linear mixed-effects models adjusting for major confounders. The secondary outcome, binary ASD incidence, will be evaluated via Firth’s penalized logistic regression with exact confidence intervals (Table 1). Given the approximately 4:1 male-to-female ratio in ASD prevalence, exploratory sex-specific analyses will evaluate differential intervention effects.

Longitudinal data will be analyzed via mixed-effects models to leverage repeated observations over time. Additionally, Bayesian statistical modeling will estimate posterior probability distributions for the effect of electromagnetic exposure reduction, a robust methodological approach for limited sample sizes. Collectively, these strategies will identify early developmental divergences and generate the preliminary epidemiological evidence required to justify larger, multi-center trials.

Anticipated attrition was estimated with reference to comparable Italian newborn cohort studies. The NASCITA, NINFEA/Piccolipiù birth cohorts anticipate a 20% loss to follow-up by 24 months, extrapolated from prior experience with the low-burden, passive data-collection protocols embedded in routine pediatric care used by the NINFEA and Piccolipiù cohorts [72]. Active behavioral interventions over a comparable postnatal window have reported considerably higher attrition [73]. Given the higher behavioral burden associated with sustained household EMR-shielding compliance over the same 24-month window, an attrition rate of 20–22% is assumed. Missing data across longitudinal assessments will be addressed using multiple imputation by chained equations (MICE), under a missing-at-random assumption; primary analyses will be repeated as complete-case and available-case sensitivity analyses.

### 2.6 Safety Considerations

The Faraday canopy is a passive, grounded, silver-coated net with no active electrical or electromagnetic emission. Canopy design will comply with standard infant sleep-safety requirements regarding ventilation and entanglement prevention. The ethics committee review (Ethics and Dissemination) will specifically evaluate the canopy’s physical safety profile for infants as an explicit approval criterion prior to enrollment.

## 3. Discussion

The present study protocol translates the Resonant Convergence framework [57] into a prospective epidemiological investigation, shifting from genetic determinism to a dynamic Gene-Environment interaction paradigm to test whether EMR acts as a modulatory cofactor in ASD pathogenesis via ICR detuning of the Ca^2+^-CaM pathway. Although this framework offers coherent multi-scale integration, its applicability to infant connectome development remains theoretically grounded; this pilot study is therefore conceived as an initial *in vivo* test of these biophysical models, not as their presupposition. Crucially, its core public health utility rests on the empirical testing of exposure reduction — independent of ICR/TR validation — as a significant neurodevelopmental divergence between cohorts would constitute a medically vital endpoint irrespective of mechanistic confirmation.

The following sections discuss the expected impact of a positive finding and the inherent methodological limitations.

### 3.1 Expected Study Impact

If the primary hypothesis is supported, this study will yield substantial theoretical and clinical impact. From a bio-physiopathological perspective, as it would provide the first prospective *in vivo* validation of the ICR and Thermomagnetic Resonance (TR) models applied to neurodevelopment. Demonstrating that low-intensity, non-thermal EMFs alter early neuronal synchronization and MNS maturation would validate the hypothesis of electromagnetic epigenetic modulation and support the development of a novel EMR safety protocol for newborns. Specifically, it would confirm the *in vivo* up-regulation of ASD-linked genes (e.g., TBR1, FOXG1) via BET-dependent mechanisms, paralleling recent observations in human cortical organoids [35].

Secondly, from a clinical and public health standpoint, identifying EMR as an actionable, non-chemical environmental trigger during the critical first 2 years would introduce a new primary prevention strategy. Standardized sleep-phase electromagnetic shielding and proactive dosimetric reduction could become highly cost-effective, low-burden, non-pharmacological recommendations for families, particularly those with a baseline genetic susceptibility to ASD, fundamentally altering pediatric environmental medicine guidelines.

### 3.2 Methodological Limitations

Despite the rigorous methodological framework, several limitations inherent to environmental epidemiology must be explicitly acknowledged:

• Observational Design and Confounding: The physical and behavioral nature of household shielding precludes double-blind randomization; a credible sham-shield is technically unfeasible, and mandating this intervention against parental preference over 24 months would drive high, differential non-adherence. Allocation is thus observational, based on parental choice, making cluster-randomized or stepped-wedge designs impractical. Multivariable regression and Bayesian models will adjust for measured baseline confounders (socioeconomic status, parental age, perinatal history), though residual confounding from unmeasured environmental co-exposures (e.g., toxicants) may persist. To detect residual bias, we incorporate two negative controls: ambient sleep-room temperature (negative exposure) and 24-month height-for-age percentile (negative outcome). Lacking randomization, the primary analysis adopts a Target Trial Emulation framework [74], setting time zero at birth and comparing sustained sleep-phase EMR-reduction against standard exposure over 24 months. Time-varying exposure status—validated monthly via sensors and dosimeters—and covariates are used to construct monthly inverse probability of treatment weights (IPTW) and inverse probability of censoring weights (IPCW) to address differential attrition. These dynamic weights feed marginal structural models (MSMs) estimating the per-protocol effect of EMR reduction. Finally, an E-value sensitivity analysis [75] will quantify the minimum strength of association an unmeasured confounder must have with both exposure and outcome to explain away the observed risk ratio. Ultimately, these methods adjust only for measured, time-varying covariates; unmeasured confounding and differential diagnostic bias remain acknowledged design limitations.

• Dosimetric Complexity and Practical Feasibility: Continuous ambient monitoring, embedded shield-integrity sensors, and wearable dosimeters (see Exposure Control and Environmental Monitoring) provide objective, multi-layered exposure verification, yet quantifying cumulative near-field exposure accurately over 24 months remains technically challenging. A related risk is behavioral: canopy compliance is expected to decline as infants gain nighttime mobility during the second year of life, and sensor hardware may itself degrade or fail over this period. Quarterly in-home verification visits and monthly cross-checks against wearable dosimetry are designed to detect both forms of degradation and prompt corrective action (equipment replacement, re-training); nonetheless, residual under-detection of declining shield fidelity cannot be fully excluded.

• Statistical Power Limits: With an expected cohort of 1000 subjects and a baseline ASD prevalence of approximately 2.76% [34], the study is intrinsically designed as an exploratory pilot. A Fisher’s exact test power calculation (Monte Carlo simulation, 20,000 iterations, n = 500/arm, two-sided α = 0.05) indicates that statistical power to detect relative risk reductions of 30%, 50%, 70%, and 80% is approximately 10.3%, 27.0%, 57.2%, and 75.0%, respectively (Table 1). The study is therefore underpowered even for large effect sizes and is explicitly designed for exploratory signal detection rather than confirmatory causal inference, carrying a non-trivial risk of Type II error. Accordingly, this pilot is designed primarily to generate effect-size and variance estimates for the formal power calculation of a future, adequately powered multi-center confirmatory trial, rather than to support definitive clinical conclusions in its own right.

## 4. Conclusions

Autism Spectrum Disorder (ASD) is characterized by complex alterations in neural connectivity, electrophysiological synchronization, and cellular metabolism. While Mirror Neuron System (MNS) dysfunction underlies the core social and cognitive deficits [4,5,6], systemic metabolic disturbances—specifically mitochondrial dysfunction, oxidative stress, and altered calcium signaling—constitute the foundational pathophysiology [14,15,16,39,40]. The exponential increase in anthropogenic electromagnetic radiation during the critical first 24 months of neurodevelopment represents a ubiquitous environmental variable directly interacting with these sensitive biological systems.

Applying the Resonant Convergence framework [57], we postulate that exogenous electromagnetic perturbations, mitochondrial redox imbalance, and consequent thermodynamic chaos synergistically disrupt the precise neural timing essential for MNS maturation. The prospective cohort study presented herein provides a structured methodological framework for the first empirical epidemiological investigation of this hypothesis.

Ultimately, characterizing these bio-electromagnetic interactions is critical for advancing two major clinical frontiers: the implementation of early primary prevention strategies—such as targeted environmental sleep-phase shielding—and the development of novel, biologically calibrated neuromodulation therapies designed to restore physiological neural synchronization in ASD.

## Figures and Tables

**Table A1.** **Synaptic function and neuronal signaling genes**.

Gene	Biological function & established ASD relevance	Potentially modulating ELF-EMF frequency
SHANK3	Postsynaptic scaffolding protein organizing glutamatergic receptor complexes; mutations are a well-established cause of core ASD phenotypes [79].	10–20 Hz
NRXN1/NLGN3/4	Trans-synaptic adhesion molecules coordinating pre/post-synaptic alignment; disrupted in ASD cohorts [80].	8–10 Hz
CNTNAP2	Neurexin-family protein regulating cortical circuit assembly and language-related neuronal migration [81].	1–3 Hz

ELF-EMF, extremely low-frequency electromagnetic field.

**Table A2.** **Genes involved in inflammatory response and oxidative stress**.

Gene	Biological function & established ASD relevance	Potentially modulating ELF-EMF frequency
MET	Receptor tyrosine kinase regulating interneuron migration, immune development, and cortical connectivity; disrupted MET signaling is documented in ASD [82].	2 Hz, 50 Hz
PTEN	PI3K/AKT/mTOR-pathway phosphatase; Pten haplo-insufficiency produces macrocephaly, oxidative stress, and mitochondrial dysfunction alongside autistic-like social deficits in animal models [83].	4 Hz, 50 Hz
SOD1/2	Antioxidant enzymes; reduced activity is documented in ASD, consistent with oxidative stress as a pathophysiological feature [84].	4 Hz

PI3K, phosphoinositide 3-kinase; AKT, protein kinase B.

**Table A3.** **Circadian rhythm and sleep regulation genes**.

Gene	Biological function & established ASD relevance	Potentially modulating ELF-EMF frequency
CLOCK/BMAL1	Core circadian transcriptional regulators; altered expression is associated with the sleep-architecture disturbances common in ASD [85].	1 Hz
MECP2	Epigenetic chromatin regulator essential for synaptic maturation; loss-of-function causes Rett syndrome, including severe sleep-wake rhythm disruption [86].	1–3 Hz

**Table A4.** **Mitochondrial function and energy metabolism genes**.

Gene	Biological function & established ASD relevance	Potentially modulating ELF-EMF frequency
SLC25A12	Mitochondrial aspartate/glutamate carrier (AGC1) regulating calcium-dependent metabolic homeostasis; overactivity is documented in ASD subgroups [87].	4 Hz, 50 Hz
OXPHOS	Electron transport chain complexes; hypofunction is documented in ASD alongside pro-inflammatory microglial shift [88].	4 Hz

OXPHOS, oxidative phosphorylation.

**Table A5.** **Neurotransmitter system genes**.

Gene	Biological function & established ASD relevance	Potentially modulating ELF-EMF frequency
SLC6A4	Serotonin transporter; variants are linked to hyperserotonemia and altered social-reward timing in ASD [89].	10 Hz, 50 Hz
GABRB3	GABA-A receptor β3 subunit; downregulation is linked to reduced mu-rhythm suppression and cortical hyperexcitability in ASD [90].	3 Hz, 12 Hz
ADORA2A/3	Adenosine receptors modulating microglial activation and neuroinflammatory pathways [71]	75 Hz

## Data Availability

This article describes a prospective study protocol; no datasets have been generated or analyzed at this stage. Upon completion of the study, data supporting the findings will be made available in a publicly accessible repository, subject to compliance with participant privacy, informed consent restrictions, and applicable data protection regulations.

## Abbreviations

ASD, Autism Spectrum Disorder; MNS, Mirror Neuron System; EMR, electromagnetic radiation; VGCCs, Voltage-Gated Calcium Channels; ICR, Ion Cyclotron Resonance; RF, radiofrequency; ELF, extremely low frequency; ADOS-2, Autism Diagnostic Observation Schedule, Second Edition; ADI-R, Autism Diagnostic Interview-Revised; DSM-5, Diagnostic and Statistical Manual of Mental Disorders, Fifth Edition; EEG, electroencephalogram; BET, bromodomain and extraterminal protein; ANS, autonomic nervous system; HRV, heart rate variability; ROS, reactive oxygen species; IFG, Inferior Frontal Gyrus; IPL, Inferior Parietal Lobule; STS, Superior Temporal Sulcus; STDP, spike-timing-dependent plasticity; NMDA, N-methyl-D-aspartate; cAMP, cyclic adenosine monophosphate; CREB, cAMP response element-binding protein; CaM, calmodulin; CaMKII, calcium/calmodulin-dependent protein kinase II; MCU, mitochondrial calcium uniporter; TR, thermomagnetic resonance; RR, risk ratios; ATP, adenosine triphosphate; ESCS, Early Social Communication Scales; CDI, MacArthur-Bates Communicative Development Inventories; MSEL, Mullen Scales of Early Learning; SHAI, Short Health Anxiety Inventory.

## Appendix A

This appendix provides the thermodynamic and quantum-electrodynamic formalism underlying the Resonant Convergence model’s account of ICR/TR loss (A.1–A.2), together with the complete candidate gene-frequency association tables referenced in Materials and Methods (A.3).

**A.1 Thermodynamic formalism**

Within the nonequilibrium thermodynamic framework proposed by Lucia [21,22,23], living systems continuously dissipate energy while maintaining ordered functional states. In this model, cellular resonance frequency is directly proportional to the rate of entropic dissipation and inversely proportional to the cell’s density, specific heat, and effective radius — linking cellular energy dissipation, metabolic activity, and electromagnetic responsiveness. Within the Resonant Convergence model, loss of ICR tuning is conceptualized as a shift toward increased entropic dissipation driven by mitochondrial dysfunction and oxidative stress, rather than as a change in the induced field itself, whose magnitude (Eqn. 1, main text) remains far too small to directly perturb ion orbits.

**A.2 Thermal-noise constraint and contested transduction hypotheses**

Within the Resonant Convergence framework, the incompatibility between weak-field Lorentz forces and physiological thermal noise (kT) — first formalized by Adair, who showed the discrepancy spans roughly ten orders of magnitude and renders the predicted ICR orbital radius incompatible with cellular dimensions [67,68,69] — is addressed, not dismissed, through two explicitly Tier-3 hypotheses: collective QED coherence domains, proposed to shield a weak ionic signal from thermal decoherence via N^(3/2) collective scaling [76,77], and the cascade-amplification mechanism described in the main text for the analogous Eqn. 1 magnitude gap [57]. Both remain contested — the coherence-domain hypothesis has been directly challenged on theoretical grounds [78] — and neither is presented as established.

**A.3 Candidate gene-frequency associations**

Candidate genes are grouped according to their principal biological functions, including synaptic signaling (Table A1, Ref. [79,80,81]), inflammatory and oxidative-stress pathways (Table A2, Ref. [82,83,84]), circadian regulation (Table A3, Ref. [85,86]), mitochondrial metabolism (Table A4, Ref. [87,88]), and neurotransmitter systems (Table A5, Ref. [71,89,90]). The tables below summarize the candidate genes, their biological functions, and the frequency ranges hypothesized to modulate their expression based on the Resonant Convergence/ICR framework [57]. The candidate genes included in Tables A1,A2,A,A4,A5 have established or proposed relevance to synaptic organization, cortical connectivity, oxidative stress, circadian regulation, mitochondrial function, and neurotransmitter signaling in ASD.
